# TREM2-directed therapy in Alzheimer’s disease: from therapeutic window to clinical translation

**DOI:** 10.3389/fnagi.2026.1938062

**Published:** 2026-09-15

**Authors:** Qi Kuang, Shujun Li, Wendi Huang, Nanyu Kuang, Tingting Liu, Nanqu Huang, Yong Luo, Juan Huang

**Affiliations:** 1 Key Laboratory of Basic Pharmacology and Joint International Research Laboratory of Ethnomedicine of Ministry of Education, Zunyi Medical University, Zunyi, Guizhou, China; 2 Department of Neurology, Third Affiliated Hospital of Zunyi Medical University (The First People’s Hospital of Zunyi), Zunyi, Guizhou, China; 3 Institute of Science and Technology for Brain-Inspired Intelligence, Fudan University, Shanghai, China; 4 Neuroimaging Research Branch, National Institute on Drug Abuse, National Institutes of Health, Baltimore, MD, United States; 5 National Drug Clinical Trial Institution, Third Affiliated Hospital of Zunyi Medical University (The First People’s Hospital of Zunyi), Zunyi, Guizhou, China; 6 Guizhou Key Laboratory of Integrated Traditional Chinese and Western Medicine, Guiyang, Guizhou, China; 7 Department of Geriatrics, Third Affiliated Hospital of Zunyi Medical University (The First People’s Hospital of Zunyi), Zunyi, Guizhou, China

**Keywords:** Alzheimer’s disease, clinical translation, microglia, neuroinflammation, therapeutic window, TREM2

## Abstract

Alzheimer’s disease (AD) is characterized by amyloid-β (Aβ) deposition, tau pathology, synaptic dysfunction, and a sustained neuroimmune response. Among immune-related targets, triggering receptor expressed on myeloid cells 2 (TREM2) is of particular interest because it is supported by human genetics, microglial biology, and expanding therapeutic development. TREM2 regulates microglial survival, phagocytosis, lipid handling, metabolic fitness, and plaque-associated responses, yet its therapeutic significance is more complex than a simple protective receptor model suggests. Although multiple TREM2-directed strategies have entered preclinical and early clinical development, recent evidence indicates that pharmacological target engagement does not necessarily translate into clinical benefit. We therefore propose that the therapeutic value of TREM2 is best understood through the concept of therapeutic window. TREM2 modulation is more likely to be beneficial when amyloid pathology is still being actively contained and microglial functional reserve remains preserved, whereas later disease stages, tau-associated neurodegeneration, receptor shedding, genetic heterogeneity, and pre-existing immune dysfunction may narrow or alter treatment benefit. Within this framework, soluble TREM2 (sTREM2) should be interpreted cautiously, as it may reflect receptor shedding, target engagement, microglial state, disease stage, or a combination of these processes rather than serving as a direct surrogate of efficacy. Viewed in this way, the central challenge of TREM2-directed therapy is to determine both when receptor modulation can still produce meaningful tissue protection and how the mode of receptor engagement shapes adaptive or maladaptive microglial programs.

## 1 Introduction

Alzheimer’s disease (AD) is one of the leading causes of dementia worldwide and is characterized by progressive cognitive decline together with a complex pathological cascade involving amyloid-β (Aβ) deposition, tau pathology, synaptic dysfunction, and a sustained neuroimmune response (Jack et al., 2018; Scheltens et al., 2021; Aarsland et al., 2025). Despite substantial advances in disease biology and biomarker-based diagnosis, effective disease-modifying therapy remains limited. Although therapeutic development has long centered on Aβ and tau, the modest and often inconsistent clinical translation of these approaches has increased interest in immune mechanisms that shape disease progression and therapeutic responsiveness (Cummings, 2023; Yadollahikhales and Rojas, 2023; Chen, X. et al., 2026).

Among immune-related targets, triggering receptor expressed on myeloid cells 2 (TREM2) has attracted particular attention because it is supported simultaneously by human genetics, microglial biology, and expanding therapeutic development (Hashioka et al., 2020; Serradas et al., 2024; Shi et al., 2025). TREM2 is highly enriched in microglia within the central nervous system and is closely linked to the sensing of injury-associated signals, phagocytosis, cell survival, metabolic adaptation, and inflammatory regulation (Qin et al., 2021; Qi et al., 2025; Chen, W. et al., 2026). These functions place TREM2 at a critical intersection between amyloid-associated injury, microglial state transitions, and the broader neuroinflammatory environment of AD. On this basis, multiple TREM2-directed strategies have entered preclinical or early clinical development, including agonistic antibodies (Wang et al., 2020; Long et al., 2024; Sharma and Singh, 2026), small-molecule agonists (Mirescu et al., 2024; Cho et al., 2025; Yuan et al., 2025), and other emerging approaches (Zhao et al., 2022c; Vandermeulen et al., 2024).

However, the therapeutic implications of TREM2 are more complex than a simple protective receptor model would suggest. Accumulating evidence indicates that TREM2-dependent responses vary with disease stage, pathological background, genetic variation, receptor shedding, and the pre-existing state of microglia (Schlepckow et al., 2017; Qin et al., 2021; Qiao et al., 2023). In early amyloid pathology, TREM2-related signaling is generally associated with plaque containment and adaptive microglial responses (Wang et al., 2016; Gandy and Ehrlich, 2023; Zhong et al., 2023). In contrast, in later or more complex disease settings, including tau-associated neurodegeneration, the biological consequences of receptor modulation may become less predictable and may no longer remain uniformly beneficial (Zhang, W. et al., 2023). This context dependence is central to understanding why strong mechanistic plausibility has not yet translated into equally strong clinical efficacy.

Recent therapeutic experience has sharpened this issue. The AL002 program showed that pharmacological engagement of TREM2 in humans is feasible and can produce measurable pharmacodynamic changes, yet these signals were not accompanied by clear clinical benefit in symptomatic AD (Wang et al., 2020; Alector, 2024; Inc, 2025). This discrepancy does not negate TREM2 as a therapeutic target. Rather, it suggests that the major challenge lies in defining when and in whom TREM2 modulation is likely to be beneficial, which modes of receptor engagement can induce adaptive microglial responses, and by what biological criteria those responses should be interpreted. In particular, biomarkers such as soluble TREM2 (sTREM2) may reflect receptor shedding, microglial activation state, disease stage, target engagement, or a combination of these processes, and therefore should not be treated as direct surrogates of efficacy (Del-Aguila et al., 2019; Biel et al., 2023; Zhang et al., 2025).

This review does not treat TREM2 as a uniformly beneficial target whose activity should simply be increased. Instead, we integrate the therapeutic window hypothesis with emerging evidence that TREM2 signaling output varies with ligand context, receptor clustering, downstream pathway coupling, and the functional state of microglia. Accordingly, we re-examine TREM2 biology, disease-stage dependence, biomarker complexity, and current therapeutic programs, with particular attention to both the timing of intervention and the mode of signaling modulation required to promote tissue-protective rather than maladaptive microglial responses.

## 2 TREM2 as a context-dependent regulator of microglial state

### 2.1 Core signaling architecture of TREM2

TREM2 is a single-pass transmembrane receptor of the immunoglobulin superfamily that is highly enriched in microglia within the central nervous system (Jay et al., 2017; Belsare et al., 2022). Structurally, it contains an extracellular immunoglobulin-like domain, a transmembrane region with a charged lysine residue, and a short cytoplasmic tail that lacks intrinsic signaling motifs (Qin et al., 2021; Chen, W. et al., 2026). As a result, intracellular signaling depends on adaptor proteins rather than direct signaling by the receptor itself (Peng et al., 2010). This structural feature is important because it places TREM2 within a broader signaling network whose biological output depends on ligand availability, adaptor coupling, and the pre-existing functional state of microglia, rather than on receptor abundance alone.

The extracellular domain of TREM2 interacts with a broad range of ligands, including lipids, phosphatidylserine, apolipoproteins, and other damage-associated molecules (Qin et al., 2021; Qi et al., 2025). In AD-related settings, reported ligands include APOE, Aβ-associated lipid signals, galectin-3, and sphingosine-1-phosphate (Nguyen et al., 2020; Xue et al., 2022). This diversity is often interpreted as broad damage perception; however, the assumption that the binding of all ligands triggers the same intracellular program is mechanistically implausible. APOE isoforms bind to TREM2 with varying affinities, driving microglial transcriptomic signatures associated with lipid metabolism and plaque compaction (Wang et al., 2015; Yeh et al., 2016; Kober et al., 2017). Specifically, APOE engagement impairs the function of the CD163-high microglial subpopulation, delays Aβ clearance, and exacerbates AD pathogenesis (Nguyen et al., 2020). In contrast, the recognition of phosphatidylserine primarily facilitates apoptotic clearance via partially overlapping but distinct downstream pathways, while galectin-3 binding amplifies pro-inflammatory cascades and aggravates neuroinflammation (Boza-Serrano et al., 2019).

Importantly, evidence for ligand-dependent divergence in TREM2 output is not limited to endogenous ligands. TREM2 has been identified as a receptor for non-glycosylated mycolic acids from mycobacteria, and in that context receptor engagement dampens anti-mycobacterial macrophage activation, indicating that TREM2 can function as a brake on innate immune responses depending on the ligand and biological setting (Iizasa et al., 2021). Conversely, the synthetic sulfoglycolipid Sulfavant A (SULF A) has been reported as a selective TREM2 ligand that promotes a homeostatic immunoregulatory program after receptor engagement (Gallo et al., 2022). Notably, a subsequent study in AD models further showed that this class of synthetic TREM2-targeting glycolipid can enhance microglial clearance, reduce amyloid burden, confer neuroprotection, and improve cognitive performance (Gallo et al., 2025). Taken together, these findings argue against a simple receptor-occupancy model and instead support the concept that distinct ligands may bias TREM2 toward different functional outputs according to their biochemical properties, mode of receptor engagement, and cellular context.

At present, it remains unresolved whether these differences arise from ligand-specific receptor conformations, altered clustering geometry at the membrane, differential coupling to DAP12/SYK-PI3K/PLCγ2 signaling modules, or context-dependent integration with the pre-existing microglial state. Until these structure–function relationships are clarified, treating all TREM2 ligands as interchangeable activators will limit both mechanistic interpretation and the rational design of ligand-selective therapeutic strategies.

Following ligand engagement, TREM2 primarily transmits signals via the DAP12 and DAP10 adaptor complex (Peng et al., 2010). DAP12 contains an immunoreceptor tyrosine-based activation motif (ITAM) that, upon phosphorylation, recruits spleen tyrosine kinase (SYK), leading to the initiation of downstream signaling cascades (Mecca et al., 2018; Ennerfelt et al., 2022; Wang, S. et al., 2022). DAP10, on the other hand, has been implicated in PI3K-dependent signaling in certain experimental contexts, particularly in pathways related to cell survival and metabolism (Sakaguchi et al., 2014). Together, these pathways link TREM2 activation to cytoskeletal remodeling, calcium signaling, phagocytic activity, and metabolic activation (Mecca et al., 2018; Ennerfelt et al., 2022; Wang, S. et al., 2022). Consequently, TREM2 signaling is more accurately described as operating predominantly through the DAP12-SYK axis, with additional modulation from DAP10-PI3K coupling, rather than depicting SYK as an upstream activator of adaptor molecules.

The canonical DAP12-SYK-PLCγ2 signaling cascade provides a simplified representation of a more complex and nuanced signaling architecture. Signal output is fine-tuned by multi-layered regulatory mechanisms, such as incomplete DAP12 phosphorylation. Under these conditions, a hidden ITIM may emerge, allowing the recruitment of the inhibitory phosphatase SHIP1, which attenuates PI3K-dependent signaling (Peng et al., 2010; Jesudason et al., 2023). Additionally, the scaffold protein LAB recruits the E3 ubiquitin ligase Cbl, which downregulates Src and SYK activity (Colonna, 2023). This regulatory complexity underscores that TREM2 signaling functions as a tunable system rather than a simple binary on–off switch, with its ultimate output determined by factors such as receptor clustering, phosphorylation stoichiometry, and the balance between activating and inhibitory signals.

Ligand-dependent signaling bias has emerged as a key concept that challenges the linear receptor-effector model. For instance, the TREM2 variant T96K, initially classified as a gain-of-function mutation based on its enhanced signaling *in vitro*, paradoxically impairs microglial activation and disease-associated microglial responses *in vivo* (Penati and Colonna, 2026; Pilat et al., 2026). This paradox highlights that enhanced signaling observed in simplified systems does not necessarily predict functional outcomes in complex tissue environments. It also suggests that different modes of receptor engagement, whether via distinct ligands, antibody epitopes, or binding valency, may preferentially activate specific downstream pathways, producing effects that cannot be inferred from aggregate measures of receptor phosphorylation alone.

Phospholipase C gamma 2 (PLCγ2) functions primarily as a downstream effector of the TREM2-DAP12-SYK pathway (Peng et al., 2010; Zhou et al., 2018). Earlier studies demonstrate that PLCγ2 facilitates calcium mobilization, phagocytic responses, and microglial survival downstream of receptor activation. More recent work in AD models suggests bidirectional crosstalk in which PLCγ2 deficiency reduces TREM2 expression and impairs plaque-associated microglial responses (Messenger et al., 2025). This feedback-like coupling supports the view that TREM2 operates within an adaptive signaling network rather than as part of a linear receptor-effector cascade.

### 2.2 TREM2 in microglial survival, phagocytosis, metabolism, and inflammatory adaptation

Functionally, TREM2 is closely linked to microglial survival, chemotaxis, phagocytosis, and the acquisition of disease-responsive states in neurodegeneration (McQuade et al., 2020; Zhou et al., 2020; Wang, S. et al., 2022). In amyloid-related settings, TREM2 deficiency impairs plaque association, weakens plaque compaction, and disrupts microglial adaptation to pathological stress (Yuan et al., 2016). These findings are often summarized as evidence that TREM2 is protective, but a more precise interpretation is that TREM2 helps preserve microglial competence under conditions of chronic injury.

This competence extends beyond phagocytosis alone. TREM2-related signaling supports lipid handling, metabolic fitness, and the coordinated reorganization of microglial responses around sites of pathology (Feiten et al., 2026). When TREM2 function is impaired, microglia may become less capable of managing lipid-rich debris, less effective at maintaining local plaque-associated barriers, and less able to sustain an ordered adaptive response. In this sense, TREM2 does not simply increase or suppress inflammation. Rather, it shapes the quality, localization, and functional efficiency of microglial activation.

This distinction is important for therapeutic interpretation. If TREM2 is viewed only as a receptor that promotes beneficial activation, then stronger stimulation might appear inherently desirable. However, the available evidence supports a more conditional model. TREM2-directed signaling appears most relevant when microglia still retain sufficient functional plasticity to mount an organized response to pathological stress. Once that reserve is substantially reduced, receptor engagement alone may be insufficient to restore beneficial function. This stage-dependent view becomes central when TREM2 is considered as a therapeutic target rather than merely a microglial marker.

### 2.3 Genetic variation and context-dependent receptor function

The biological importance of TREM2 in AD is strongly reinforced by human genetics. Multiple coding variants have been linked to altered receptor function and disease susceptibility, among which R47H is the best established example (Guerreiro et al., 2013; Jonsson et al., 2013). This variant has been associated with increased AD risk and impaired microglial responses to amyloid pathology, including weaker plaque association, reduced plaque compaction, and diminished clearance-related function (Guerreiro et al., 2013; Jonsson et al., 2013; Baligács et al., 2024; Yu et al., 2025). These findings support the view that compromised TREM2 function can shift microglia toward a less adaptive state in the presence of accumulating pathology.

TREM2 variants do not exert a single uniform effect. H157Y provides a more complex example. Located near the TREM2 shedding site, this variant has been associated with increased sTREM2 production (Schlepckow et al., 2017; Qiao et al., 2023). In experimental amyloid models, H157Y has been linked to reduced amyloid pathology and altered synaptic function (Qiao et al., 2023). However, more recent evidence suggests that its effects are not uniformly protective and may also be associated with greater neurodegeneration and altered immune-related processes in certain disease contexts (Tsui et al., 2025). These observations indicate that TREM2 variant biology is shaped not only by receptor signaling strength, but also by shedding dynamics, pathological background, and disease stage.

For translation, this heterogeneity has direct implications. It suggests that TREM2-directed therapy is unlikely to show uniform effects across genetically diverse patient populations and that genotype may influence not only disease risk but also treatment response, pharmacodynamic interpretation, and safety profile (Ma et al., 2025). A framework that ignores this layer of biological variation is therefore unlikely to capture the full therapeutic potential or limitation of TREM2 modulation.

### 2.4 Membrane-bound TREM2 and sTREM2 as linked but non-equivalent regulators

sTREM2 arises from at least two sources, proteolytic shedding of membrane-bound TREM2 and alternative splicing of TREM2 transcripts (Schlepckow et al., 2017; Del-Aguila et al., 2019). Shedding is mediated mainly by ADAM10 and ADAM17 near the H157-S158 region, releasing the extracellular domain into the extracellular space (Schlepckow et al., 2017). In addition, transcript-specific studies have shown that a TREM2 isoform lacking exon 4, which encodes the transmembrane domain, may also contribute to the sTREM2 pool (Del-Aguila et al., 2019). This dual origin is important because it means that sTREM2 cannot be interpreted simply as a one-dimensional readout of receptor activation.

In AD research, sTREM2 is most commonly discussed as a cerebrospinal fluid biomarker related to microglial activation (Schauer et al., 2024; Zhang et al., 2025). CSF sTREM2 levels increase during symptomatic stages and are associated with neuronal injury markers and microglia-related responses (Zhang et al., 2025). In individuals with early Aβ abnormalities, higher CSF sTREM2 has also been associated with amyloid-related increases in p-tau and cerebral glucose hypermetabolism (Biel et al., 2023). However, these associations should not be overinterpreted as evidence that sTREM2 directly tracks therapeutic benefit or harm in a linear way.

More importantly, sTREM2 may reflect several processes at once, including receptor shedding, microglial activation state, disease stage, and the balance between membrane retention and soluble release (Schlepckow et al., 2017; Biel et al., 2023; Zhang et al., 2025). It may therefore provide useful biological information while still remaining an imperfect surrogate for treatment efficacy. In addition, experimental evidence suggests that sTREM2 is not merely a passive cleavage product, but may itself influence microglial survival, inflammatory responses, and protein aggregation under specific conditions (Zhong et al., 2017). Current evidence therefore supports a dual view. Membrane-bound TREM2 and sTREM2 are biologically linked, but they are not functionally equivalent. This distinction becomes especially important when biomarker changes are used to interpret the effects of TREM2-directed therapy.

These biological features suggest that the therapeutic significance of TREM2 cannot be defined independently of disease stage. The same receptor system may support adaptive microglial responses in one pathological context yet show limited or altered benefit in another. This stage dependence provides the basis for considering TREM2 through the concept of therapeutic window.

## 3 Therapeutic window of TREM2 across AD progression

### 3.1 Early amyloid pathology and plaque-associated microglial adaptation

Some of the strongest current evidence for a beneficial role of TREM2 comes from amyloid-related stages in which plaque deposition is emerging and microglia still retain sufficient plasticity to mount an organized local response. In this setting, TREM2 is closely involved in plaque sensing, microglial recruitment, and plaque-associated barrier formation (Wang et al., 2016; Zhong et al., 2018; Magno et al., 2021). Upon activation of the TREM2-associated signaling network, including the DAP12-SYK-PLCγ2 axis, microglia undergo cytoskeletal remodeling and functional changes that support migration, plaque association, and phagocytic responses (Magno et al., 2021). In mouse models of amyloid pathology, TREM2 deficiency impairs microglial clustering around plaques and is associated with greater plaque spread and increased neurotoxic exposure of surrounding neurites (Wang et al., 2016; Condello et al., 2018).

During early plaque formation, TREM2-responsive microglia can extend processes around amyloid deposits to form a protective cellular barrier that helps limit the diffusion of soluble toxic Aβ species (Condello et al., 2018; Park et al., 2024). As plaques mature, TREM2 is also linked to the acquisition of disease-associated microglial states and to the upregulation of lysosomal and lipid-handling programs involved in plaque-associated adaptation (Lin et al., 2024). These observations support the view that TREM2 contributes not only to clearance-related function, but also to the spatial organization and metabolic coordination of microglial responses around pathological lesions.

This early amyloid phase is where the concept of therapeutic window becomes most persuasive. When plaque-associated microglia remain functionally competent, enhancing TREM2-related signaling is more likely to support containment of amyloid-associated injury and preservation of local tissue homeostasis. However, these effects should not be reduced to a single uniform pathway of Aβ recognition. Rather, they reflect a broader adaptive response whose benefit depends on the timing of intervention and the residual capacity of microglia to respond efficiently. Recent work also suggests that externalized phosphatidylserine associated with injured neurons may facilitate TREM2-dependent recognition of Aβ-related damage signals (Park et al., 2024), further indicating that the relevance of TREM2 in this stage lies in coordinated injury sensing rather than in a simple receptor-ligand model.

In addition to membrane-bound TREM2, sTREM2 may also influence amyloid-related processes. *In vitro* studies indicate that sTREM2 interacts preferentially with fibrillar rather than monomeric Aβ and inhibits secondary nucleation, thereby reducing the generation of new fibrillar species (Belsare et al., 2022). Although these findings do not justify treating sTREM2 as a direct therapeutic mediator in AD, they do suggest that the biological impact of TREM2 in early amyloid disease may involve both membrane signaling and soluble receptor dynamics. Importantly, the overall impact of TREM2 signaling on Aβ pathology appears to be stage dependent. In early amyloid deposition, enhanced TREM2 activity is generally associated with improved plaque containment and clearance-related responses, whereas prolonged or dysregulated activation in later disease settings may contribute less consistently to beneficial outcomes (Gandy and Ehrlich, 2023). This stage dependence is central to the therapeutic timing of TREM2-directed intervention (Figure 1).

**Figure 1:**
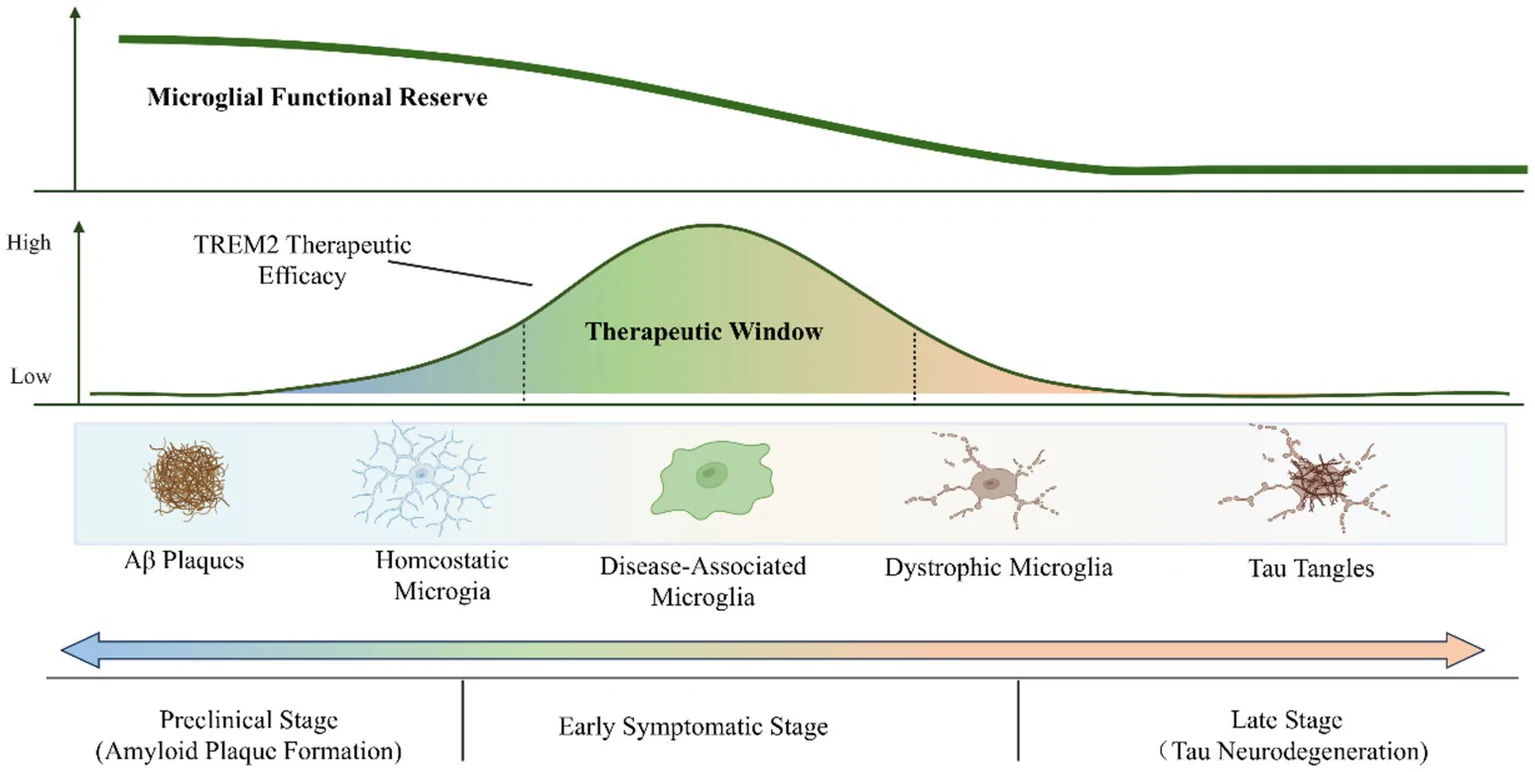
Therapeutic window and signaling context of TREM2-directed therapy across AD progression. This conceptual model illustrates that the potential efficacy of TREM2-directed therapy varies with disease stage and microglial functional competence. The biological outcome also depends on the mode of receptor engagement and whether downstream signaling is coupled to adaptive, tissue-protective microglial responses. The curves and stage boundaries are schematic and do not represent quantitatively established clinical relationships.

### 3.2 Tau-associated neurodegeneration and the narrowing therapeutic window

Compared with amyloid pathology, the role of TREM2 in tau-driven neurodegeneration appears more context dependent and should be interpreted with greater caution (Huang et al., 2023). Increasing evidence indicates that TREM2 influences microglial responses to tau-related injury and may modulate the local environment in which tau pathology develops and propagates (Zhang, X. et al., 2023; Chen et al., 2025; Li et al., 2025). However, the direction and magnitude of these effects vary across models and are less uniformly supportive of simple therapeutic activation.

Experimental studies suggest that TREM2 can affect microglial containment and clearance of pathological tau, as well as the inflammatory and metabolic responses associated with tauopathy (Bemiller et al., 2017; Chen et al., 2025). In tau transgenic models, TREM2 deficiency has been associated with aggravated tau hyperphosphorylation, broader dysregulation of neuronal stress kinase pathways, and worsened neurodegenerative changes (Bemiller et al., 2017). Conversely, microglia-specific elevation of TREM2 has produced only modest benefits, including reductions in soluble phosphorylated tau and limited preservation of neuronal integrity, without clearly reducing insoluble tau burden (Chen et al., 2025). These findings suggest that TREM2-related signaling may influence tau pathology, but its effects are unlikely to be uniformly protective across all disease stages and pathological settings.

The interpretation becomes even more complex when plaque context and APOE background are considered. Prior studies have suggested that TREM2-dependent microglial responses may help restrict tau seeding in plaque-associated settings (Li et al., 2025), whereas other work indicates that tau-mediated neurodegeneration can still proceed in the setting of microglial activation that is not fully dependent on TREM2, particularly in the presence of APOE ε4 (Gratuze et al., 2023). It is therefore more accurate to state that TREM2 shapes microglial responses to tau pathology rather than to conclude that it exerts a single direct and uniformly suppressive effect on tau spread.

These observations suggest that the therapeutic window of TREM2 may narrow as disease shifts from predominantly amyloid-associated injury toward more advanced tau-associated neurodegeneration. At this stage, microglia may already be metabolically constrained, functionally exhausted, or embedded in a pathological environment that is less responsive to receptor-centered modulation. Under such conditions, receptor engagement may still be detectable, but the probability that it will restore meaningful tissue protection becomes less certain (Dias and Socodato, 2025; Zhang et al., 2025). This distinction is highly relevant to clinical development, because a strategy that is biologically plausible in early amyloid disease may be applied too late in symptomatic tau-dominant stages.

In addition to membrane-bound TREM2, sTREM2 may also participate in tau-related regulation. One recent study reported that sTREM2 can bind neuronal transgelin-2 and inhibit the RhoA-ROCK-GSK3β pathway, thereby reducing tau hyperphosphorylation in experimental models (Zhang, X. et al., 2023). Although this mechanism is intriguing, it should currently be presented as a specific experimental finding rather than as a broadly established mechanism of tau control in AD. Overall, the available evidence supports a modulatory role for TREM2 in tau pathology, but one whose therapeutic relevance is more contingent and stage sensitive than in early amyloid-associated disease.

### 3.3 Synaptic protection, inflammatory homeostasis, and metabolic fitness

Beyond its roles in amyloid and tau pathology, TREM2 also contributes to synaptic protection and the regulation of inflammatory homeostasis in the AD brain. Recent evidence has suggested that TREM2 may interact with complement component C1q and may thereby influence classical complement activation linked to synaptic loss (Zhong et al., 2023). In this context, TREM2 may help preserve synaptic integrity not only through phagocytic clearance, but also by restraining maladaptive inflammatory amplification around vulnerable synapses.

TREM2 shapes the inflammatory profile of microglia through several downstream pathways. In general, TREM2 activation has been associated with microglial survival, metabolic support, and a more balanced immune response, whereas TREM2 deficiency or dysfunction tends to impair these adaptive responses and promote inflammatory dysregulation (Cianciulli et al., 2020; Colonna, 2023). However, these effects should not be simplified into a fixed binary model of anti-inflammatory versus pro-inflammatory signaling. A more accurate view is that TREM2 modulates the magnitude, timing, and quality of microglial responses in a context-dependent manner.

An important component of this regulatory role is metabolic fitness. TREM2-related signaling supports lipid handling, mitochondrial function, and the energetic demands required for effective microglial responses (Ulland et al., 2017). When TREM2 function is impaired, microglia can develop metabolic stress, lysosomal dysfunction, and reduced capacity to maintain tissue homeostasis, changes that may secondarily aggravate inflammatory injury and synaptic vulnerability (Ulland et al., 2017). In parallel, TREM2 interacts functionally with other innate immune pathways, including receptors involved in lipid sensing and phagocytosis such as CD36 (Kim et al., 2017). These observations suggest that TREM2 acts within a broader immune-metabolic network rather than as an isolated receptor.

This broader perspective helps explain why the therapeutic window of TREM2 cannot be defined solely by plaque burden or tau stage. It is also shaped by whether microglia retain sufficient metabolic reserve and regulatory capacity to translate receptor modulation into coordinated protective function. In earlier disease stages, TREM2-related responses are more often associated with plaque containment, synaptic protection, and adaptive microglial activation (Wang et al., 2016; Zhong et al., 2023). As disease advances, the consequences of TREM2 signaling become increasingly dependent on pathological burden, microglial state, and the integrity of the surrounding immune-metabolic environment (Zhang, W. et al., 2023).

### 3.4 When TREM2 signaling becomes insufficient or uncoupled from benefit

Current evidence supports a dynamic role for TREM2 across AD progression rather than a uniformly beneficial one. In earlier stages, especially when amyloid pathology is emerging and microglial adaptation remains preserved, TREM2-related signaling is more likely to support plaque containment, synaptic protection, and local inflammatory balance (Wang et al., 2016; Gandy and Ehrlich, 2023; Zhong et al., 2023). As disease advances, this beneficial window may narrow for several reasons. The pathological landscape becomes more complex, tau-associated degeneration becomes more prominent, receptor biology may be altered by shedding or genotype, and microglia may enter states in which additional receptor engagement no longer produces proportional functional benefit.

This does not mean that TREM2 becomes simply harmful in late disease. Rather, it suggests that TREM2-directed intervention may become biologically insufficient, clinically ineffective, or constrained by the broader state of the diseased tissue. This distinction is important. A lack of therapeutic efficacy should not automatically be interpreted as evidence that TREM2 is irrelevant. It may instead indicate that intervention has occurred outside the most responsive therapeutic window, or that the measured pharmacodynamic changes do not adequately capture beneficial microglial reprogramming.

Accordingly, the therapeutic promise of TREM2 lies less in the assumption that receptor activation is inherently beneficial and more in identifying both when microglia remain responsive and which modes of receptor engagement can elicit adaptive rather than maladaptive programs. This integrated view of therapeutic timing and signaling quality helps explain why biological plausibility does not always translate into consistent therapeutic effects.

## 4 Biomarker complexity in TREM2-directed therapy

### 4.1 Interpretive complexity of sTREM2 as a biomarker

Among the biomarkers currently discussed in TREM2-directed therapy, sTREM2 has received the greatest attention, but also poses the greatest interpretive difficulty. sTREM2 is commonly regarded as a cerebrospinal fluid biomarker related to microglial activation, and this use is biologically reasonable to a certain extent (Schauer et al., 2024; Zhang et al., 2025). However, sTREM2 should not be treated as a direct surrogate of target engagement or therapeutic efficacy. Its biological meaning is inherently composite because it can arise from proteolytic shedding of membrane-bound TREM2 as well as from alternative splicing of TREM2 transcripts (Schlepckow et al., 2017; Del-Aguila et al., 2019). As a result, any change in sTREM2 may reflect altered receptor shedding, altered membrane receptor availability, altered microglial state, or a combination of these processes.

This complexity becomes even more important in the AD setting because the relationship between sTREM2 and disease activity is itself stage dependent. CSF sTREM2 levels increase during symptomatic stages and have been associated with markers of neuronal injury and microglial response (Schauer et al., 2024; Zhang et al., 2025). In individuals with early Aβ abnormalities, higher sTREM2 has also been associated with amyloid-related increases in p-tau and cerebral glucose hypermetabolism (Biel et al., 2023). Yet these associations do not establish whether sTREM2 is marking adaptive compensation, incomplete immune containment, or a biologically mixed state in which both processes coexist. For this reason, sTREM2 should be interpreted as a context-sensitive indicator of TREM2-related biology rather than as a linear measure of therapeutic success.

A further complication is that sTREM2 may not be only a biomarker. Experimental evidence suggests that it can also influence microglial survival, inflammatory responses, and protein aggregation under certain conditions (Zhong et al., 2019). This means that membrane-bound TREM2 and sTREM2 are biologically linked but not functionally interchangeable. A biomarker that also has biological activity cannot be interpreted in the same way as a passive pharmacokinetic readout. In the context of drug development, this distinction is critical because it weakens any simplistic assumption that more or less sTREM2 necessarily indicates more or less therapeutic benefit (Figure 2).

**Figure 2:**
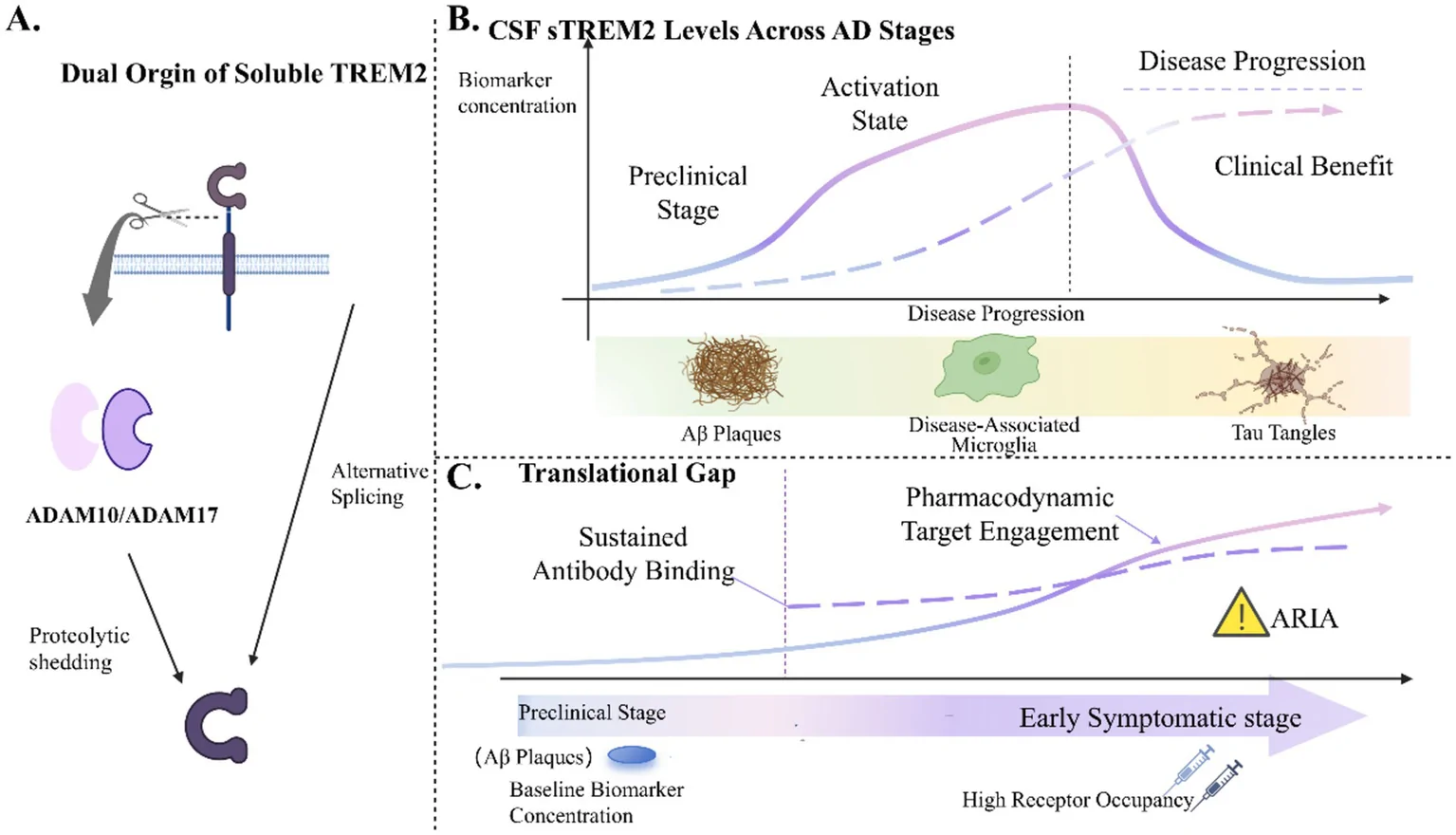
Biomarker complexity and translational gap in TREM2 therapy. **(A)** sTREM2 may arise from proteolytic shedding of membrane-bound TREM2 or alternative transcript processing. **(B)** CSF sTREM2 varies across AD stages but does not necessarily parallel clinical benefit. **(C)** Pharmacodynamic target engagement may occur without clinical efficacy. ARIA, amyloid-related imaging abnormalities. All curves are schematic and do not represent quantitatively established clinical relationships.

### 4.2 Target engagement versus beneficial microglial reprogramming

One of the central challenges in this field is the tendency to blur the distinction between pharmacodynamic engagement and beneficial disease modification. This problem is especially visible in TREM2-directed therapy, where measurable biomarker shifts can occur without corresponding clinical improvement. In principle, biomarkers related to TREM2 modulation can be divided into at least two categories. The first category includes markers that indicate target engagement or receptor-proximal biological activity, such as changes in sTREM2 or other immune-related soluble factors. The second includes markers that more directly reflect beneficial microglial reprogramming or disease-relevant downstream effects, such as preserved plaque containment, improved synaptic integrity, reduced neurodegenerative injury, or ultimately clinical stabilization (Schlepckow et al., 2023; Ma et al., 2025; Wang et al., 2026).

These two categories are not equivalent. A treatment may clearly engage TREM2 or alter receptor shedding while still failing to restore the broader microglial functions required for meaningful tissue protection. This distinction is particularly important because TREM2 signaling operates within a wider immune-metabolic network. Even when receptor-proximal activity is achieved, the downstream cellular response may remain constrained by disease stage, genetic background, microglial exhaustion, or the surrounding pathological environment. In other words, a biomarker can confirm that a drug is biologically active without proving that the biological activity is sufficient, well-timed, or functionally beneficial.

For this reason, future studies should avoid treating a single pharmacodynamic marker as a stand-alone indicator of efficacy. A more rigorous interpretation requires asking whether the observed biomarker change captures receptor engagement only, or whether it also corresponds to improved microglial organization, preserved metabolic competence, and better coupling between immune response and tissue protection. Without this distinction, TREM2-directed development risks overestimating the significance of early biomarker signals.

### 4.3 Integrating CSF, plasma, imaging, and genotype-informed readouts

Because no single biomarker fully captures the therapeutic meaning of TREM2 modulation, interpretation is likely to require integrated readout systems rather than reliance on one analyte alone. CSF sTREM2 can provide information about TREM2-related biology within the central nervous system, but its meaning remains inseparable from receptor shedding and disease stage (Schlepckow et al., 2017; Zhang et al., 2025). Additional fluid biomarkers may help contextualize this signal. For example, changes in markers related to microglial activation, neurodegeneration, or inflammatory tone may clarify whether a shift in sTREM2 is occurring in parallel with broader biological adaptation or in isolation from it (Pesämaa et al., 2023).

Imaging biomarkers may provide a different layer of information. In preclinical and translational settings, TSPO-PET and FDG-PET have been used to assess microglial activity and cerebral metabolic responses after TREM2-directed intervention (van Lengerich et al., 2023). Although these modalities are themselves not free of interpretive limitations, they may help bridge the gap between soluble pharmacodynamic changes and spatially resolved brain responses. Likewise, amyloid PET and tau-related measures may be useful not simply as end-stage disease markers, but as tools for defining whether treatment is being applied within a biologically plausible therapeutic window.

Genotype-informed interpretation is also likely to become increasingly important. TREM2 variants can alter receptor function, shedding, and downstream adaptation, while APOE background can modify how microglial responses intersect with amyloid and tau pathology (Giorgio et al., 2025). A biomarker profile that is informative in one genetic background may therefore be less informative in another. Taken together, these considerations suggest that future TREM2-directed trials will likely require composite biomarker strategies that integrate fluid biomarkers, imaging, and genetic stratification rather than relying on a single molecular signal.

### 4.4 Biomarker interpretation across disease stages and therapeutic modalities

Biomarker meaning in TREM2-directed therapy is not fixed across disease stages or treatment classes. In earlier amyloid-dominant disease, a biomarker pattern consistent with increased plaque-associated microglial adaptation may carry a different implication than a superficially similar pattern observed in later symptomatic disease, where tau-associated injury, synaptic loss, and broader tissue dysfunction already predominate. The same biomarker shift may therefore indicate adaptive immune engagement in one context and biologically insufficient compensation in another. This stage dependence is one reason why biomarker interpretation should be anchored to therapeutic window rather than treated as biologically self-explanatory.

The treatment modality also matters. Antibodies that activate TREM2, antibodies that reduce receptor shedding, small molecules that alter receptor conformation or shedding dynamics, and indirect modulators of related pathways may all influence overlapping biomarkers through partly different mechanisms (Schlepckow et al., 2020; van Lengerich et al., 2023; Cho et al., 2025). As a result, similar changes in sTREM2 or related markers should not automatically be assumed to reflect the same underlying biology across modalities. A reduction in sTREM2 after one intervention may reflect reduced shedding and increased membrane retention, whereas under another condition it may simply mark altered receptor processing without clear functional gain.

These issues become particularly important in clinical translation, where biomarker shifts are often among the earliest measurable signs of drug activity. The central task is therefore not merely to detect change, but to interpret what kind of change has occurred and whether it is likely to matter biologically. A more mature biomarker framework for TREM2-directed therapy should distinguish receptor engagement from effective microglial reprogramming, and short-term pharmacodynamic activity from disease-relevant benefit. Without that distinction, even well-executed trials may generate biomarker signals that are biologically interesting but therapeutically ambiguous.

For therapeutic programs, the key question is not only whether TREM2-directed agents alter biomarker profiles, but whether those changes meaningfully inform the interpretation of efficacy, timing, and mechanism in clinical development.

## 5 Lessons from TREM2-directed therapeutic programs

### 5.1 Agonistic antibodies as proof of pharmacological tractability

Among currently available TREM2-directed strategies, agonistic antibodies have provided the clearest evidence that the receptor can be pharmacologically engaged *in vivo*. Their importance lies not only in their therapeutic promise, but also in the fact that they have served as biological tests of whether microglial TREM2 can be modulated in a way that is both feasible and disease relevant. Preclinical studies have largely supported this possibility. Across several antibody programs, TREM2 activation has been associated with enhanced microglial phagocytic activity, altered plaque morphology, reduced amyloid-related burden, and in some settings improvement in synaptic or behavioral readouts (van Lengerich et al., 2023; Mummery et al., 2026). These findings helped establish TREM2 as a druggable microglial receptor rather than merely a genetic or pathological correlate of disease.

At the same time, the antibody field has also shown that receptor activation is not a biologically uniform intervention. Some antibodies primarily enhance signaling, some reduce extracellular domain shedding while also activating the receptor, and others incorporate engineering strategies to improve avidity or brain delivery (Schlepckow et al., 2020; van Lengerich et al., 2023; Kraller et al., 2025). For this reason, antibody-based programs are most informative not simply because they activate TREM2, but because they reveal how strongly translational outcome depends on the mode of receptor engagement. This point becomes most evident in the AL002 program.

### 5.2 AL002 as a translational test case

The AL002 program illustrates this translational problem particularly clearly. AL002 is a humanized agonistic monoclonal antibody developed to activate TREM2 and enhance microglial responses in AD (Wang et al., 2020; Sharma and Singh, 2026). Its development built on related antibodies including AL002a and AL002c, which helped establish proof of concept across mouse and human TREM2 systems (Long et al., 2024; Sharma and Singh, 2026). In preclinical studies, these agents were associated with reduced Aβ deposition, altered plaque morphology, and enhanced microglial activity, supporting the view that receptor activation could produce biologically meaningful effects in amyloid-bearing models (Long et al., 2024; Sharma and Singh, 2026).

The translational significance of AL002 lies in what happened next. In the Phase I INVOKE-1 study, AL002 showed measurable cerebrospinal fluid exposure together with pharmacodynamic changes consistent with biological activity, including reductions in CSF sTREM2 and an increase in soluble colony-stimulating factor 1 receptor (sCSF1R) (Long et al., 2024). These results were important because they demonstrated that TREM2-directed engagement in humans is feasible and that receptor-centered pharmacology can be detected *in vivo*. However, the subsequent Phase II INVOKE-2 study did not demonstrate clinical benefit. Although AL002 achieved sustained target engagement and biomarker shifts, it did not meet the primary endpoint and did not show significant benefit across major secondary clinical outcomes, and biomarker changes were not accompanied by convincing improvement in neurodegeneration-related or amyloid-related measures (Alector, 2024; Inc, 2025; Mummery et al., 2026).

This outcome provides several important lessons. First, target engagement is necessary but clearly insufficient. A drug can modify TREM2-related biology without delivering meaningful clinical benefits, particularly in symptomatic AD. Second, while the pharmacodynamic markers used in the study were biologically informative, they did not clarify whether the treatment induced beneficial microglial reprogramming or merely receptor-proximal activity. Third, the failure of AL002 should not be attributed solely to a restricted therapeutic window. Mechanistically, several alternative explanations merit consideration. Antibody-based agonists may engage TREM2 in a non-physiological manner, leading to signaling bias. For instance, they might preferentially activate the SYK pathway without adequately engaging the PI3K-Akt or PLCγ2 branches, thereby inducing dysfunctional microglial responses (Peng et al., 2010; Kober and Brett, 2017). Furthermore, excessive or prolonged TREM2 stimulation may result in receptor desensitization or maladaptation of downstream signaling, particularly in late-stage AD, where microglia are already in a dysfunctional or exhausted state (Krasemann et al., 2017; Ulland et al., 2017). Thus, the negative result of AL002 may reflect a mismatch between the intensity of receptor activation and the functional competence of microglia, rather than a temporal limitation alone.

In summary, AL002 should not be dismissed simply as a failed program. Instead, it should be regarded as an important translational case study, demonstrating that TREM2 biology can be targeted in humans while highlighting the need for a careful reassessment of factors such as treatment timing, patient selection, biomarker strategies, and signaling modulation.

### 5.3 Shedding-modulating and engineered antibodies

Beyond AL002, preclinical antibody programs have been informative not because they constitute a single class of equivalent agonists, but because they test different translational solutions to the same problem, preserving membrane TREM2, strengthening receptor clustering, and improving brain delivery. One important line of work has focused on antibodies that reduce TREM2 extracellular domain shedding while preserving membrane-associated receptor levels. The best example is 4D9, which targets the ADAM10 and ADAM17 cleavage region in the stem domain of TREM2 and thereby decreases extracellular shedding while also promoting receptor activation through bivalent binding (Schlepckow et al., 2020). *In vitro* and *in vivo* studies showed enhanced microglial phagocytosis together with reduced amyloid-related measures, supporting the idea that preserving membrane TREM2 may be therapeutically advantageous. However, because 4D9 does not recognize human TREM2, its main value lies in mechanistic proof of concept rather than direct clinical translatability (Schlepckow et al., 2020).

A second important line has focused on engineering for avidity and brain delivery. Ab2 and Ab18 were reformatted into tetravalent dual-variable-domain immunoglobulin designs that markedly enhanced agonistic activity and strengthened phagocytosis-, migration-, and survival-related responses (Zhao et al., 2022a; Zhao et al., 2022b). These studies showed that antibody format itself can be a major determinant of biological effect, which is highly relevant in a system where signaling output depends on receptor clustering and downstream pathway engagement. More advanced delivery-oriented designs extended this logic further. ATV: TREM2 and its murine analog ATV:4D9 used transferrin receptor-based transport to improve brain exposure and were associated with enhanced *in vivo* biological responses, including higher microglial activity and altered metabolic signals on imaging (van Lengerich et al., 2023). M07-TFN similarly combined TREM2 agonism, reduced extracellular domain shedding, and transferrin receptor 1-mediated transport, with reported reductions in plaque burden and cognitive benefit in 5 × FAD mice (Kraller et al., 2025).

The broader lesson from these programs is that translational success may depend not only on whether TREM2 is activated, but on whether the mode of activation is compatible with sufficient central target engagement, balanced signaling intensity, and acceptable safety. Engineering strategies that improve central exposure or stabilize membrane TREM2 may help address some of the limitations seen with conventional antibodies. However, these approaches also introduce new layers of complexity, including altered pharmacology, potential peripheral effects, and uncertainty about how much biological amplification is actually desirable in later-stage disease. Their promise therefore lies in expanding the design space of TREM2 modulation, not yet in resolving the translational problem.

### 5.4 Small-molecule agonists and emerging non-antibody approaches

Non-antibody approaches are important less because they have already solved the translational problem and more because they test whether TREM2 biology can be modulated with greater pharmacological flexibility than antibodies allow. In principle, small molecules offer several attractive advantages, such as oral dosing, easier medicinal chemistry optimization, and potentially better central nervous system exposure (Mirescu et al., 2024; Cho et al., 2025; Yuan et al., 2025). This field remains early, but it has already produced representative compounds including VG-3927, the first small-molecule TREM2 agonist to enter clinical evaluation, as well as newer candidates such as C1 and AS48 (Mirescu et al., 2024; Cho et al., 2025; Yuan et al., 2025).

What these compounds have shown so far is encouraging but still preliminary. C1 improved on earlier scaffolds by offering a clearer dose-dependent profile, direct if modest binding to TREM2, and better *in vitro* pharmacokinetic properties (Yuan et al., 2025). AS48 added a mechanistically interesting feature by combining receptor agonism with inhibition of extracellular domain shedding, thereby potentially preserving membrane-associated TREM2 while also promoting downstream signaling (Cho et al., 2025). However, the current evidence base for these agents remains limited by modest binding affinity, incomplete *in vivo* validation, and insufficient long-term characterization of selectivity, pharmacokinetics, and safety (Cho et al., 2025; Yuan et al., 2025). At present, the small-molecule field shows that TREM2 is not restricted to antibody-based pharmacology, but it has not yet demonstrated that these compounds can solve the biological and translational challenges identified by antibody programs.

Other emerging strategies broaden the field further by acting at the level of RNA regulation or pathway derepression rather than direct receptor agonism. A ribonuclease H-active antisense oligonucleotide targeting TREM2 RNA showed biological activity in human induced pluripotent stem cell-derived systems and altered microglia-related inflammatory responses *in vivo*, but did not produce clear improvement in amyloid plaque burden (Vandermeulen et al., 2024). This suggests that transcript-level modulation of TREM2 is feasible, yet its disease-modifying significance remains uncertain. Similarly, the LILRB2-blocking antibody Ab29 was developed to relieve inhibitory pressure on TREM2-related signaling rather than activate TREM2 directly. In preclinical settings, Ab29 partially restored microglial phagocytosis and migration, supporting the concept that convergent inhibitory pathways may be therapeutically relevant (Zhao et al., 2022c). These strategies are valuable because they show that TREM2-directed therapy does not need to be limited to simple agonism. Even so, their current role is primarily exploratory.

### 5.5 What preclinical success has and has not predicted

Across genetic and pharmacological studies, increasing TREM2 expression or enhancing receptor activity has been shown to modulate microglial responses, reduce amyloid-related pathology, and, in some AD mouse models, improve cognitive performance (Jiang et al., 2014; van Lengerich et al., 2023; Long et al., 2024). In this sense, the preclinical literature has successfully shown that TREM2 is a biologically relevant and druggable target.

However, preclinical success has been less effective in predicting which biological signals will translate into meaningful benefit in patients. It has not reliably defined how late in the disease course receptor modulation remains useful, which pharmacodynamic changes reflect beneficial microglial reprogramming rather than receptor-proximal activity, or how genotype and baseline microglial state shape treatment response (Wang et al., 2020; Alector, 2024; Inc, 2025; Mummery et al., 2026). This limitation is not surprising, since many preclinical studies rely on amyloid-dominant or relatively early disease models that may overrepresent the conditions under which TREM2 activation is most effective. By contrast, symptomatic human AD typically involves broader pathological complexity, including tau-associated injury, synaptic loss, and chronic tissue dysfunction.

The key lesson, therefore, is not that preclinical studies failed to identify TREM2 as a meaningful target. Rather, they have been less successful in predicting when, in whom, and through which mode of receptor modulation target engagement will translate into clinically relevant benefit. This gap defines the central translational problem addressed in the following section.

## 6 Why target engagement has not yet translated into clinical benefit

### 6.1 Disease stage and timing of intervention

One plausible explanation for the current translational gap is that TREM2-directed therapy may be most effective only within a restricted biological window. Preclinical evidence has consistently shown that TREM2 supports plaque-associated microglial adaptation, phagocytic responses, and local tissue protection in early amyloid-related disease (Wang et al., 2016; Gandy and Ehrlich, 2023). However, symptomatic human AD is not simply an amplified version of early amyloid pathology. By the time patients enter many clinical trials, tau-associated neurodegeneration, synaptic loss, circuit dysfunction, and broader tissue damage are already well established (Jack et al., 2010; Mondragón-Rodríguez et al., 2020). Under these conditions, even a biologically active TREM2-directed intervention may be acting too late to restore a level of microglial coordination sufficient to produce measurable clinical benefit.

This stage dependence also helps explain why strong mechanistic effects in amyloid-dominant models have been difficult to reproduce clinically. Many preclinical systems capture a disease phase in which plaque-associated microglia remain relatively plastic and pathology is still amenable to immune-supported containment. In contrast, symptomatic AD represents a more heterogeneous and biologically advanced condition. The issue, therefore, may not be that TREM2 is an invalid target, but that the intervention is frequently deployed after the most responsive therapeutic window has already narrowed. This interpretation is consistent with the observation that pharmacodynamic activity can still be detected in patients even when downstream clinical benefit remains absent (Wang et al., 2020; Alector, 2024; Inc, 2025).

For future development, this point carries a practical implication. TREM2-directed therapy should not be framed simply as a general immune intervention for established AD. Rather, its clinical value may depend heavily on earlier deployment, more precise stage definition, and better alignment between the dominant pathology and the specific microglial functions that TREM2 modulation is expected to influence (Wang et al., 2026).

### 6.2 Baseline microglial state and pathological context

A second major issue is that TREM2 signaling does not operate in a biologically neutral environment. Its effect depends on the baseline state of microglia and on the pathological context in which receptor modulation occurs. In early disease, when microglia still retain functional reserve, TREM2 activation may help preserve plaque containment, metabolic adaptation, and local inflammatory balance (Wang et al., 2016; Ulland et al., 2017; Qin et al., 2021). In later disease, however, microglia may already be metabolically stressed, transcriptionally dysregulated, or functionally exhausted. In that setting, receptor engagement alone may not be sufficient to restore coordinated protective responses.

This problem is particularly relevant because TREM2 does not control a single isolated process. It influences survival, lipid handling, phagocytosis, inflammatory tone, and state transitions within a broader immune-metabolic network (Ulland et al., 2017; Zhou et al., 2018; Wang, S. et al., 2022). If the surrounding pathological environment has already shifted toward severe tau-associated injury, synaptic degeneration, and chronic tissue stress, then the downstream consequences of TREM2 activation may be attenuated, biologically mixed, or simply too limited to change clinical trajectory. In other words, target engagement may occur in a system whose response capacity is already compromised.

The anti-human TREM2 antibody (hT2AB) study provides a useful conceptual example. The biological effect of anti-TREM2 treatment varied according to pre-existing microglial activation state, sex, and genetic background, and did not translate into clear evidence of reduced overall plaque burden or direct therapeutic benefit (Ellwanger et al., 2021). Although preclinical, this finding supports a broader principle. The response to TREM2-directed therapy is likely conditioned by the microglial state present before treatment begins. Without accounting for that baseline heterogeneity, clinical trials may pool together patients whose immune systems differ substantially in their capacity to respond.

### 6.3 Beyond receptor activation: signaling rewiring as a translational objective

The importance of baseline microglial state also points to a broader issue in TREM2-directed therapy. If the functional consequences of TREM2 modulation depend on the pre-existing competence of microglia, then the central translational question is not only whether the receptor can be engaged, but whether that engagement is converted into a biologically useful downstream program. In this sense, therapeutic failure may reflect not only treatment outside the optimal disease window, but also an inability to induce the appropriate signaling state.

This distinction is increasingly relevant because TREM2 should not be viewed as a receptor with a fixed and uniform output. Its signaling consequences are shaped by ligand context, receptor organization, proteolytic shedding, downstream pathway coupling, and the cellular state in which engagement occurs (Ulland and Colonna, 2018; Schlepckow et al., 2020; Qin et al., 2021). TREM2 interacts with multiple ligands, including phosphatidylserine, APOE-containing lipoproteins, Aβ-associated lipid assemblies, and galectin-3, yet it remains uncertain whether these distinct forms of engagement are functionally equivalent at the intracellular level (Atagi et al., 2015; Bailey et al., 2015; Zhao et al., 2018; Boza-Serrano et al., 2019). Likewise, although canonical signaling through DAP12, SYK, PI3K, and PLCγ2 is well established, available data suggest that the biological output of TREM2 cannot be inferred from receptor engagement alone (Peng et al., 2010; Sims et al., 2017; Ulland et al., 2017; Ennerfelt and Lukens, 2020).

From this perspective, future therapeutic development may need to move beyond the assumption that stronger receptor activation is necessarily preferable. In some settings, the more relevant objective may be to favor specific downstream outputs, such as metabolic adaptation, plaque containment, or tissue-protective microglial responses, while avoiding forms of activation that are non-physiological, poorly coupled to disease-relevant effector function, or unsustainable in later-stage disease. The emerging concept of TREM2 signaling rewiring therefore refines rather than replaces the therapeutic window framework. It suggests that a meaningful therapeutic window depends not only on disease stage, but also on whether microglia remain capable of translating receptor modulation into an adaptive and clinically relevant response program (Schlepckow et al., 2017; Ennerfelt and Lukens, 2020; Wang, M. et al., 2022).

This view also has implications for the interpretation of clinical programs. A negative outcome with a TREM2-directed agent should not be understood solely as evidence that treatment was initiated too late. It may also indicate that pharmacological engagement was not matched to the signaling configuration required for beneficial microglial reprogramming. For this reason, future development will likely require greater distinction between receptor-proximal pharmacodynamic activity and downstream evidence of effective state transition in microglia (Ulland and Colonna, 2018; Ennerfelt and Lukens, 2020; Schlepckow et al., 2020).

### 6.4 Genetic background and inter-individual heterogeneity

Genetic heterogeneity is another likely contributor to the disconnect between target engagement and efficacy. TREM2 variants can alter receptor signaling, shedding dynamics, and downstream adaptation, while other AD-relevant genetic factors, particularly APOE background, can reshape how microglial responses interact with amyloid and tau pathology (Schlepckow et al., 2017; Gratuze et al., 2023; Qiao et al., 2023; Tsui et al., 2025). These influences are unlikely to be biologically trivial. They may affect both the baseline role of TREM2 in a given patient and the way a TREM2-directed therapy is interpreted at the level of biomarkers, tissue response, and safety.

This issue has two dimensions. First, not all patients start from the same receptor biology. A therapeutic strategy designed around the common form of TREM2 may not operate in the same way in individuals carrying functional variants affecting ligand interaction or shedding. Second, the downstream tissue context shaped by other genetic factors may alter whether TREM2 activation remains adaptive, becomes insufficient, or carries higher risk. The association between higher amyloid-related imaging abnormality rates and APOE ε4 homozygosity in the AL002 program illustrates that genotype may influence not only efficacy but also tolerability (Alector, 2024; Inc, 2025; Mummery et al., 2026).

From a translational perspective, these observations argue against a uniform treatment model. If TREM2-directed therapy is evaluated without adequate genetic stratification, true biological signals may be diluted across heterogeneous subgroups, while safety liabilities may become harder to interpret (Ma et al., 2025; Feiten et al., 2026). More selective trial enrichment based on TREM2 and APOE background may therefore be needed, not because these genes explain everything, but because they directly influence the biology the therapy is trying to modulate.

### 6.5 Drug delivery, receptor occupancy, and safety trade-offs

Even when the target and disease stage are reasonably selected, translational success still depends on whether the drug reaches the brain in a biologically meaningful way and whether sufficient receptor modulation can be achieved without unacceptable safety cost. This is particularly challenging for antibody-based TREM2 therapies. Large molecules typically face limited central nervous system penetration, which means that central exposure and effective target engagement in the brain may remain limited unless dosing is intensified or delivery is actively engineered (Zhao et al., 2022b; van Lengerich et al., 2023). Yet stronger exposure does not automatically solve the problem. More aggressive immune modulation may increase the risk of adverse effects, especially in vulnerable patient subgroups.

This trade-off is already visible in current development programs. Engineering strategies such as transferrin receptor-mediated delivery have improved brain exposure and strengthened *in vivo* biological responses in preclinical models (van Lengerich et al., 2023; Kraller et al., 2025). These advances are important because they show that technical limitations of delivery are not insurmountable. However, they also highlight that improving central exposure is only one part of the problem. The more difficult question is how much receptor engagement is optimal. Too little exposure may produce only partial biological activity, while excessive or poorly timed activation may increase inflammatory burden or imaging abnormalities without improving functional outcome.

The clinical findings with AL002 reinforce this point. Sustained target engagement was accompanied by higher rates of amyloid-related imaging abnormalities and greater discontinuation in some treated groups, especially among APOE ε4 homozygotes (Alector, 2024; Inc, 2025; Mummery et al., 2026). These data suggest that the therapeutic margin of TREM2 modulation may be narrower than initially expected. Accordingly, future development will need to optimize not only whether the drug reaches the target, but whether the degree and mode of engagement remain compatible with long-term safety and disease-stage appropriateness.

### 6.6 Why pharmacodynamic readouts do not guarantee clinical benefit

Perhaps the most direct reason why target engagement has not yet translated into clinical benefit is that pharmacodynamic activity and therapeutically meaningful biological change are not the same thing. In TREM2-directed therapy, receptor engagement can be reflected by measurable biomarker shifts, including changes in sTREM2 and related immune markers (Wang et al., 2020; Alector, 2024). However, these readouts do not by themselves establish that microglia have been reprogrammed in a way that improves tissue protection, slows neurodegeneration, or preserves cognition. At best, they indicate that the drug is biologically active at or near the intended target.

This distinction is especially important because TREM2-related biomarkers are themselves biologically composite. A change in sTREM2 may reflect altered shedding, altered membrane receptor retention, altered microglial activation state, or a mixture of these processes (Schlepckow et al., 2017; Biel et al., 2023; Zhang et al., 2025). Likewise, downstream soluble markers may indicate immune engagement without revealing whether the response remains adaptive, incomplete, or potentially maladaptive. In this context, the gap between pharmacodynamic signal and clinical effect is not surprising. It reflects a deeper biological issue, namely that receptor-proximal activity may be easier to detect than disease-relevant immune remodeling.

Accordingly, future trials should place less weight on single pharmacodynamic shifts as implicit evidence of efficacy and more emphasis on integrated biological interpretation. The key question is not simply whether a marker changed, but whether the observed change is plausibly linked to improved plaque containment, preserved synaptic integrity, reduced neurodegenerative stress, or better functional outcome. Until that link is established more rigorously, TREM2-directed therapy may continue to generate biologically interesting signals that fall short of clinically meaningful benefit.

### 6.7 A working model of the translational gap

The current translational gap in TREM2-directed therapy is unlikely to reflect a single failure. More plausibly, it arises from the convergence of several factors, intervention outside the most responsive disease window, heterogeneity in baseline microglial state, genetic and biological variation, incomplete central target modulation, safety constraints, and imperfect interpretation of pharmacodynamic markers. None of these factors alone is sufficient to dismiss TREM2 as a therapeutic target. However, in combination, they help explain why strong preclinical rationale and measurable biological activity have not yet yielded clear clinical efficacy.

This interpretation supports a more restrained but more useful view of the field. What has failed so far may be less the target itself than the assumption that target engagement can substitute for stage-appropriate and functionally meaningful microglial reprogramming. Future progress will therefore depend less on simply intensifying receptor activation and more on matching TREM2-directed therapy to the right disease stage, biological context, and response framework. These considerations provide the basis for a more selective translational strategy, outlined in the next section.

## 7 Toward clinical translation of TREM2-directed therapy

### 7.1 Stage-guided patient stratification

Future clinical development must move beyond broad enrollment of symptomatic AD populations and toward stage-guided patient selection. The available evidence suggests that TREM2-directed intervention is more likely to be biologically relevant when amyloid pathology is actively evolving and microglial adaptive capacity remains sufficiently preserved (Wang et al., 2016; Gandy and Ehrlich, 2023; Zhong et al., 2023). By contrast, in later disease stages dominated by tau-associated neurodegeneration, synaptic loss, and extensive tissue dysfunction, receptor engagement may still occur without producing meaningful clinical gain (Bemiller et al., 2017; Gratuze et al., 2023; Chen et al., 2025). This implies that patient selection should be informed not only by clinical diagnosis, but also by biomarker-defined disease stage and by the dominant pathological process present at the time of treatment.

On the basis of current preclinical and early translational evidence, early symptomatic or even presymptomatic amyloid-positive populations may prove more informative than heterogeneous later-stage cohorts for testing whether TREM2 modulation can alter disease trajectory (Morenas-Rodríguez et al., 2022). Such an approach would also align more closely with the biology suggested by preclinical models, many of which capture relatively early amyloid-associated states rather than advanced multisystem degeneration. Stage-guided enrichment would not guarantee efficacy, but it would reduce one major source of biological mismatch between preclinical rationale and clinical testing.

### 7.2 Genotype-informed trial enrichment

Genetic heterogeneity should be more deliberately incorporated into trial design. TREM2 variants can affect receptor function, ligand interaction, and shedding dynamics (Guerreiro et al., 2013; Jonsson et al., 2013; Schlepckow et al., 2017). In parallel, APOE background can modify the relationship between microglial responses, amyloid pathology, tau-related injury, and treatment-associated safety signals (Schlepckow et al., 2017; Inc, 2025). These factors are likely to shape both baseline biology and treatment responsiveness, yet current trial strategies have not fully integrated them into enrichment logic.

This does not mean that all TREM2-directed studies must be restricted to narrowly defined genetic subgroups. Rather, it means that genotype should be treated as a biologically meaningful stratification variable rather than a purely descriptive baseline characteristic (Trastulla et al., 2024). At minimum, future studies should be designed to assess whether therapeutic response, biomarker shifts, and adverse-event profiles differ according to TREM2 and APOE status. In more advanced trial designs, genotype-informed enrichment may help identify subpopulations in which receptor biology remains more modifiable or in which the balance between efficacy and risk is more favorable.

### 7.3 Biomarker-guided dose and response assessment

Future TREM2-directed trials will require a more disciplined biomarker framework. sTREM2 and related pharmacodynamic markers remain useful, but they should be interpreted as indicators of TREM2-related biology rather than as stand-alone surrogates of efficacy (Del-Aguila et al., 2019; Biel et al., 2023; Zhang et al., 2025). Dose selection and response assessment should therefore be guided by biomarker panels that distinguish receptor-proximal activity from broader disease-relevant biological effects.

In practical terms, this suggests a layered approach. Fluid biomarkers can help define whether the drug has altered TREM2-related signaling or shedding, while imaging and downstream neurodegeneration-related markers may help determine whether those changes are accompanied by more meaningful biological adaptation (Wang et al., 2020; van Lengerich et al., 2023; Alector, 2024). A biomarker-guided framework should also account for stage dependence, since the same biomarker shift may not carry the same implication in early amyloid-dominant disease as in later symptomatic disease. The central objective is not simply to document change, but to establish whether the observed change is plausibly linked to improved microglial function, preserved tissue homeostasis, or a slower pathological trajectory.

### 7.4 Combination strategies and rational sequencing with other AD therapies

Given the biological position of TREM2 at the intersection of immune adaptation, plaque-associated response, and metabolic regulation, it is unlikely that TREM2-directed therapy will ultimately function as a universally sufficient monotherapy across all disease stages. A more realistic possibility is that it may prove most useful in combination with other AD-directed interventions or when deployed in a rational sequence relative to them (Ma et al., 2025). For example, if TREM2 modulation is most relevant during phases of active plaque-associated microglial adaptation, then combining or sequencing it with anti-amyloid strategies may be biologically more coherent than applying it as an isolated late-stage immune intervention (Schlepckow et al., 2023).

The rationale for combination approaches extends beyond amyloid. Because TREM2 influences the quality and coordination of microglial responses rather than directly eliminating all downstream pathology, its therapeutic value may depend on whether parallel processes such as tau propagation, synaptic injury, or vascular dysfunction are also being addressed. This does not yet justify any fixed combination paradigm, but it does suggest that future clinical development should consider TREM2 modulation as part of a broader disease-modifying strategy rather than as a stand-alone correction of neuroinflammation.

### 7.5 Unresolved questions for trial design and clinical translation

Several major questions remain unresolved and should guide the next phase of investigation. First, the optimal therapeutic window for TREM2-directed intervention has not yet been clearly defined in humans. Preclinical studies strongly support stage dependence, but the boundaries of that window in biomarker-defined AD remain uncertain. Second, the relationship between membrane-bound TREM2 and sTREM2 remains incompletely understood in the treatment setting. A change in sTREM2 may reflect reduced shedding, altered receptor availability, or broader changes in microglial state, but these possibilities are not yet cleanly separable (Schlepckow et al., 2017; Del-Aguila et al., 2019; Zhang et al., 2025).

Third, the degree of receptor activation that is biologically useful but still safe remains unclear. Experience with AL002 indicates that pharmacological engagement is feasible, yet also suggests that the therapeutic margin may be narrower than initially anticipated in some patient groups (Alector, 2024; Inc, 2025; Mummery et al., 2026). Fourth, it remains uncertain whether different therapeutic modalities, including agonistic antibodies, shedding-modulating antibodies, brain-delivery-engineered constructs, small molecules, and RNA-based approaches, converge on the same biologically relevant endpoint or instead influence partially distinct aspects of TREM2 biology (Schlepckow et al., 2020; van Lengerich et al., 2023; Vandermeulen et al., 2024; Cho et al., 2025). Finally, more work is needed to determine which biomarker changes truly indicate beneficial microglial reprogramming rather than receptor engagement alone.

Taken together, these unresolved questions point to a common requirement. Future trials will need to align five elements more explicitly than current studies have done: disease stage, baseline microglial state, genotype, mode of receptor engagement, and biomarker framework. Within such a design logic, the goal is not simply to show that TREM2 biology can be perturbed, but to determine whether it can be modulated in a manner that is appropriately timed, mechanistically suitable, biologically interpretable, and clinically meaningful (Supplementary Figure 1).

## 8 Conclusion

TREM2 has emerged as one of the most biologically compelling immune-related targets in AD because it links human genetics, microglial state regulation, and therapeutic development within a single translational framework. Current evidence supports important roles for TREM2 in microglial survival, phagocytosis, metabolic adaptation, plaque-associated responses, and the regulation of inflammatory homeostasis. At the same time, the available data indicate that TREM2 should not be viewed as a uniformly beneficial target whose activation is inherently advantageous across all stages of disease. Rather, its therapeutic relevance appears to depend on disease stage, pathological context, genetic background, receptor shedding dynamics, and the pre-existing functional state of microglia.

This perspective explains why strong mechanistic plausibility and measurable pharmacological activity have not yet translated into clear clinical benefit. A central question is no longer whether TREM2 is relevant to AD, but when its modulation remains biologically meaningful, which modes of receptor engagement favor adaptive microglial programs, and how such responses should be measured. Pharmacodynamic changes such as shifts in sTREM2 or related immune markers may indicate target engagement, but they do not by themselves establish beneficial microglial reprogramming or disease modification.

Taken together, TREM2 is best regarded as a context-dependent signaling system rather than a uniformly protective receptor target. Its clinical value will depend on matching disease stage and microglial competence with a mode of receptor engagement that induces sustained tissue-protective microglial responses rather than receptor-proximal pharmacodynamic changes alone. Within such a model, the major challenge is no longer simply to engage the target, but to do so at a point where microglial function remains redirectable and where biological activity can still be translated into meaningful therapeutic benefit.
